# Preparedness of South East Asia countries in view of monkeypox emergence: A call for action

**DOI:** 10.1016/j.lansea.2022.100074

**Published:** 2022-09-26

**Authors:** Ramadan Abdelmoez Farahat, Sudhan Rackimuthu, Tungki Pratama Umar, Javeria Arif Siddiqui, Abhigan Babu Shrestha, Mohammad Yasir Essar

**Affiliations:** a Faculty of Medicine, Kafrelsheikh University, Kafrelsheikh, Egypt; b Global Research Group (GRG), Kafrelsheikh, Egypt; c Father Muller Medical College, Mangalore, Karnataka, India; d Faculty of Medicine, Sriwijaya University, Palembang, Indonesia; e Sindh Medical College, Jinnah Sindh Medical University, Karachi, Pakistan; f M Abdur Rahim Medical College, Dinajpur, Bangladesh; g Kabul University of Medical Sciences, Kabul, Afghanistan

Since May 2022, a global outbreak of monkeypox (MPX) has emerged, with over 39417 cases from 90 countries, as of August 18, 2022.^1^ Due to this concerning situation, the World Health Organization (WHO) declared the current outbreak a Public Health Emergency of International Concern (PHEIC) on July 23, 2022.^2^ As of August 18, 2022, 14 cases of MPX (9 from India and 5 from Thailand) have been reported in the WHO South-East Asia Region (SEAR), which includes 11 member states namely Bangladesh, Bhutan, North Korea, India, Indonesia, Maldives, Myanmar, Nepal, Sri Lanka, Thailand, and Timor-Leste.^1^

MPX can be transmitted through various pathways, including direct skin-to-skin contact, respiratory droplets, sexual intercourse [predominantly homosexual, bisexual and men who have sex with men (MSM)], bodily fluid, and contact with contaminated objects.^3^ A vast spectrum of symptoms have been reported, including skin rash (primarily <10 lesions), fever, lethargy, myalgia, headache, and lymphadenopathy. Vulnerable groups include young children, pregnant women, and immunocompromised individuals, particularly those infected with the human immunodeficiency virus (HIV). Although it is generally mild, the overall case fatality rate has been reported to be 8.7%, and the basic reproduction number (R_0_) to be > 1 in MSM [i.e., Portugal (1.4), United Kingdom (1.6), and Spain (1.8)]. However, R_0_ is < 1 in other settings.^3^, ^4^, ^5^

MPX is caused by a complex linear double-strand DNA virus called monkeypox virus (MPXV), belonging to Orthopoxvirus genus, which is the same genus of variola virus, causing smallpox. Hence, the similarity in terms of clinical symptoms, genetic structure, and cross-protection of available smallpox vaccines against MPX (efficacy: 80-95%).^4^ Presently, there are two vaccines approved by the United States Food and Drug Administration (US-FDA) for MPX: ACAM2000 and JYNNEOS.^6^ ACAM2000 is a single dose vaccine available in large supply. It contains live Vaccinia virus, and is subsequently contraindicated in pregnancy, patients with HIV, and other immunocompromised individuals.^6^ Approved in 2019, JYNNEOS vaccine is administered in 2 doses and was deemed as relatively safe for use in most people.^6^ Unfortunately, there is little supply and reserve of JYNNEOS and should therefore be prioritized for those who are ineligible to receive the ACAM2000 vaccine.^7^ Antiviral drugs, like tecovirimat (TPOXX), although not widely available yet, are good alternatives for those unable to be vaccinated.^8^ In SEAR, vaccine availability is currently low, and hence ring vaccination would be an efficient and cost-effective approach.^9^^,^^10^ Ring vaccination is a strategy whereby close contacts of confirmed cases of disease are vaccinated.^10^ Although it requires rapid surveillance, ring vaccination proved beneficial in eradicating smallpox and a similar blueprint should therefore be followed for MPX.^9^

In SEAR, the overall risk of a MPX outbreak is moderate. However, there are several concerns about the possibility of MPXV spreading worldwide, especially after declaring it as PHEIC.^2^ Hence, this entails that MPX outbreak should be taken seriously. With air-travel and other modes of transport being slowly opened in view of decreasing overall threat of COVID-19 in most countries, the risk of spread of MPXV from one country to another has substantially risen. The primary challenge currently faced by the SEAR, stems from scarcity of vaccines and antivirals that is essential to treat and curb the spread of MPX. Furthermore, insufficient laboratory diagnostic infrastructures and modalities have been noticed that pose significant difficulties in detecting MPX cases in the SEAR.^11^

SEAR has been disproportionately affected by a plethora of communicable diseases, resulting in fragile healthcare systems.^12^ Exacerbated by COVID-19, and the burden of pre-existing diseases like HIV, Tuberculosis (TB) and malaria, SEAR countries have experienced a major setback in improving the quality of healthcare available to their respective populations.^12^ Consequently, further large outbreaks of MPX in SEAR countries will further strain healthcare systems, which are already on the brink of collapse. Despite significant progress in strengthening public health emergency preparedness among SEAR members, several issues have been identified during the current COVID-19 situation which may also affect the spread of the MPXV. These include undesirable emergency governance structures, poor surveillance and alert mechanisms, insufficient laboratories, disrupted health supply chains, weakened healthcare system readiness, and reduced community engagement. The leading causes identified as the cause for the above are lack of funding and financial support as well as a lack of reliable guidance for developing firm policies on health system reformation.^13^^,^^14^

Strategies to curb the spread of MPX have been widely discussed given the context of the ongoing outbreak during the COVID-19 pandemic. Several valuable recommendations and guidelines have been proposed by leading health organizations and researchers around the world which include intensifying epidemiological surveillance, strengthening health facilities and diagnostic centers, swift detection, isolation as well as contact tracing, ring vaccination for contacts and vulnerable populations, among many others.^5^^,^^15^ However, realistic implementation of these strategic measures in the SEAR region to contain the outbreak can occur only when a plethora of region-specific impediments can be successfully overcome.

First, the widely existing stigma against the gay community in several parts of the SEAR region,^16^ acts as an important hindrance to effectively engaging with MSM being the most vulnerable population, implicated in MPX transmission.^3^ Thereby, disrupting the most important mitigatory measure for early containment that is rapid contact tracing, with affected populations hesitating to disclose relevant contact-based information. Eliminating stigma and discrimination is an arduous and protracted task. However, engaging in advocacy, enhancing education, and pushing for amendments in policy and legislation from the grassroot level can help bring about change. Second, inadequate and/or misinformation about MPX among the SEAR population is another important cause for concern, with several at-risk and uneducated populations inhabiting remote areas; possibly resulting in either intentional or unintentional nonadherence to advocated preventive measures and delayed health seeking behavior. The same is also believed to be closely tied to widely varying levels of trust by SEAR populations in their respective governments, which are reflected historically by population compliance to government mandated interventions in the region. Low levels of trust have been demonstrated in governments of several SEAR countries, due to spread of misinformation (India),^17^ or low awareness and education (Nepal).^18^ Thus, we recommend launching tailored campaigns, educational programs and sharing of accurate medical content to increase public awareness about MPX and its transmission. This could help in enhancing the populations trust in their respective governments and in the preventive measures taken to curb the MPX outbreak. Third, the availability of vaccines for MPX are more readily available among most western countries than most countries of the SEAR. The need for MPX vaccine stockpiling is not currently necessary, and hence initiatives must be undertaken by relevant health organizations and governments to procure and equitably distribute MPX vaccines in the future to countries in need.^19^^,^^20^ Fourth, though strengthening genomic and epidemiological surveillance is recommended, particularly at hot-spots wherein international travel are routinely undertaken, some SEAR countries currently do not have the infrastructure to deploy such measures. Steps must also be taken to leverage important mechanisms such as The Association of Southeast Asian Nations (ASEAN) and The South Asian Association for Regional Cooperation (SAARC) to augment ongoing international co-operativity especially among countries part of the SEAR region to help disseminate and exchange accurate information, strategies, funds, and health related infrastructural aid.

Several important lessons can be learnt to help mount a better outbreak response against MPX in the future from the merits as well as pitfalls encountered by almost all countries part of the SEAR whilst combating COVID-19. Though, the risk of spread and lethality of MPX has been ubiquitously acknowledged to be lesser than COVID-19, it should be acknowledged as a significant public health threat and met with a different strategic approach. A principal cause for the devastating effect that COVID-19 has had on the SEAR can be attributed to its under-preparedness, lack of raising early significant awareness, delays in enforcing lockdowns, strict rules and regulations (such as strict quarantine and extensive contact tracing) as well as lack of robust policies and health regulations, especially during the early phases of the pandemic. Even though the efforts taken by most countries to combat the COVID-19 pandemic is to be lauded, repeated revisions, back-tracking of government decisions and inadequate medical infrastructure and supply reserves, especially in the context of vaccine procurement and distribution has played a significant role in protracting the pandemic.^21^ Care should be taken to avoid similar errors in judgement if a significant outbreak of MPX occurs in the region, by constitution of expert committees alongside overseeing bodies to ensure that enforced guidelines, rules and guidelines are strictly adhered to.

To conclude, the previously coined term “novel” for coronavirus has now more or less been reinstated by MPX, a virus that has been around for several decades. As of August 18, 2022, only 14 cases have been reported from SEAR nations. Should these meager cases be a matter of concern? Yes. To caveat, the impoverished health infrastructure and lack of awareness in these countries is highly prone to masking MPX among the myriad of endemic infectious diseases with similar signs and symptoms. Healthcare professionals and policymakers must therefore be extremely mindful of the ins and outs of the emerging MPX threat and existing COVID-19 burden.

## Contributors

RAF prepared the initial draft. RAF, SR, TPU, JAS and ABS wrote the draft. RAF, SR, and MYE reviewed the literature and edited the manuscript. All authors read and approved the final manuscript.

## Data sharing statement

All data are included in the above manuscript.

## Declaration of interests

None.
